# Operative invasiveness does not affect the prognosis of patients with non-small cell lung cancer

**DOI:** 10.1186/s12890-020-01264-x

**Published:** 2020-10-15

**Authors:** Nozomu Motono, Shun Iwai, Yoshihito Iijima, Katsuo Usuda, Hidetaka Uramoto

**Affiliations:** Department of Thoracic Surgery, Kanazawa Medical University, 1-1 Daigaku, Uchinada, Ishikawa 920-0293 Japan

**Keywords:** Operative invasiveness, Video-assisted thoracic surgery, Non-small cell lung cancer

## Abstract

**Background:**

The relationship between operative invasiveness and the prognosis in non-small cell lung cancer (NSCLC) patients who have undergone surgery has been controversial.

**Methods:**

Clinical data were analyzed for 463 NSCLC patients. Operative invasiveness was defined by wound length, operation time, and the postoperative C-reactive protein (postCRP) level. The operative approach was divided into video-assisted thoracic surgery (VATS) and thoracotomy.

**Results:**

The wound length and operation time were significantly correlated with the postCRP level (correlation coefficient (CC) = 0.39, *p* <  0.01; CC = 0.54, p <  0.01, respectively). The postCRP level in the VATS group was significantly lower than that in the thoracotomy group (12.2 mg/dl vs 20.58 mg/dl, *p* <  0.01). The relapse-free survival differed significantly based on wound length (*p* <  0.01), operation time (*p* = 0.01), CRP level (p <  0.01), and operative approach (p <  0.01). The carcinoembryonic antigen level (hazard ratio [HR], 1.58; *p* = 0.02), pathological stage (pStage) (HR, 2.57; *p* <  0.01), vascular invasion (HR, 1.95; *p* = 0.01), and preoperative CRP level (preCRP) (HR, 1.91; p <  0.01) were identified as significant prognostic factors for relapse-free survival in a multivariate analysis. Furthermore, the multivariate analysis showed that smoking history (HR, 2.36; *p* = 0.03), pStage (HR, 3.26; *p* <  0.01), and preCRP level were significant prognostic factors for overall survival.

**Conclusion:**

Preoperative CRP level was associated with poor prognosis. Although the VATS approach might be less invasive procedure for NSCLC patients, operative invasiveness does not affect the prognosis.

## Background

Video-assisted thoracic surgery (VATS) for patients with non-small cell lung cancer (NSCLC) has been widely adopted and the advantages of the VATS approach have been reported [1–7]. These reports have shown that VATS is associated with less pain, a shorter hospital stay, less reduction of the inflammatory-immune response, and the maintenance of the postoperative respiratory function in comparison to thoracotomy. Furthermore, it was reported that VATS is associated with significantly less response in C-reactive protein (CRP) and cytokine levels. Acute-phase proteins (APPs) are a series of proteins that are sensitive to inflammation and body stress including infection, surgical trauma, certain diseases and tissue damage [8, 9]. The concentrations of certain APPs, including CRP may increase markedly during stress or under pathological conditions. Levels of the CRP increase as a result of the inflammatory response to infection or tissue damage, and have been used to evaluate surgery technique, infection and pathology progress. The serum concentration of CRP was significantly higher in the open group, compared with the VATS group. Thus, the VATS approach has been considered to be less invasive [9]. However, the prognostic impact of the operative invasiveness—including the postoperative inflammatory response—in NSCLC patients is uncertain.

In the present study, we evaluated the prognostic impact of the operative invasiveness after surgery in NCSLC patients.

## Methods

### Patients

One thousand one hundred fifty-six NSCLC patients who underwent complete resection in Kanazawa Medical University between January 2002 and December 2018 were identified. Among these, 463 NSCLC patients had available data. These patients were enrolled in the present retrospective study.

Regarding the data collected, the clinical factors included the sex, age, smoking history, comorbidities, and the carcinoembryonic antigen (CEA) and preoperative CRP (preCRP) levels. The smoking history was assessed using the Brinkman index, which is calculated as the numbers of cigarettes smoked per day multiplied by the number of years for which the subject has smoked. Comorbidities were evaluated by the Charlson Comorbidity Index [11]. The perioperative factors included the wound length, operative approach, operative procedure, operation time, postoperative CRP (postCRP) level, and postoperative complications. The operative approach was divided into three categories: complete VATS (C-VATS; surgery was only performed to provide a monitoring view); hybrid VATS (H-VATS; surgery was combined with direct vision without rib spreading); and thoracotomy. Postoperative complications were categorized into five grades according to the Clavien–Dindo classification system [12]. Histological type, differentiation, lymphatic invasion (Ly), vascular invasion (V), and pathological stage (pStage) were evaluated as pathological factors.

### Statistical analyses

The correlation coefficient was evaluated by Spearman’s rank correlation coefficient. The cut-off values of factors associated with recurrence were calculated by an ROC curve analysis and prognostic analyses were performed based on these cutoff values. The cumulative survival rates were calculated by the Kaplan–Meier method, and survival curves were compared using a log-rank test. Univariate and multivariate analyses using a Cox proportional hazards model were conducted to determine the risk factors for relapse-free survival (RFS) and overall survival (OS). All statistical analyses were two-sided, and *p* values of < 0.05 were considered to indicate statistical significance. The statistical analyses were conducted using the JMP software program (Version 13.2; SAS Institute Inc., Cary, NC, USA).

## Results

### Patient characteristics

The clinicopathological characteristics of the 463 patients who were included in the present study are listed in Table 1. Two hundred eighty-one patients (61%) were men, and the median age was 68 years. The median Brinkman index was 600, the median CEA level was 3.6 ng/ml, and the median preCRP level was 0.1 ng/ml. More than half of patients had a low comorbidity index (Charlson Comorbidity Index of 0, *n* = 282; 61%).

**Table 1:** Patient characteristics

Gender (male / female)	281 (61%) / 182 (39%)
Age (y.o.), median (range)	68.4 (34–87)
Smoking index, median (range)	600 (0–3600)
CEA (ng/ml), median (range)	3.6 (0.5–236)
Charlson comorbidity index (0 / 1 / 2 / 3 / 4)	282 (61%) / 108 (23.2%) / 62 (13.4%) / 9 (2%) / 2(0.4%)
Wound length (cm), median (range)	8 (3–25)
Approach (C-VATS / Hybrid / Thoracotomy)	77 (17%) / 321 (69%) / 65 (14%)
Operative procedure (Seg / Lob / Bilob / Pneumo)	26 (6%) / 409 (88%) / 10 (2%) / 18 (4%)
Operation time (min), median (range)	230 (67–674)
Histology (Ad / Sq / AdSq / LCNEC / Large / Pleo)	350 (76%) / 91 (19.6%) / 8 (1.6%) / 9 (1.8%) / 3 (0.6%) / 2 (0.4%)
Differentiation (G1 / 2 / 3 / 4)	173 (37.4%) / 203 (43.8%) / 75 (16.2%) / 12 (2.6%)
Ly (0 / 1)	271 (58.5%) / 192 (41.5%)
V (0 / 1)	236 (51%) / 225 (49%)
pStage (IA / IB / IIA / IIB / IIIA)	225 (48.5%) / 101 (21.8%) / 48 (10.4%) / 43 (9.3%) / 46 (10%)
Preoperative CRP (ng/ml), median (range)	0.1 (0.02–14.89)
Postoperative CRP (ng/ml), median (range)	13.39 (0.66–36.28)
Clavien-Dindo grade (0 / 1 / 2 / 3a / 3b)	340 (73.4%) / 20 (4.3%) / 45 (9.7%) / 53 (11.4%) / 5 (1.2%)

*CEA* carcinoembryonic antigen, *VATS* video-assisted thoracic surgery, *Seg* segmentectomy, *Lob* lobectomy, *Bilob* bilobectomy, *Pneumo* pneumonectomy, *Ad* adenocarcinoma, *Sq* squamous cell carcinoma, *AdSq* adenosquamous cell carcinoma, *LCNEC* large cell neuroendocrine cell carcinoma, *Large* large cell carcinoma, *Pleo* pleomorphic cell carcinoma, *G* grade, *Ly* lymphatic invasion, *V* vascular invasion; *pStage* pathological stage, *CRP* C-reactive protein

### Perioperative factors

The operative approach was C-VATS in 77 patients (17%), H-VATS in 321 (69%), and thoracotomy in 65 (14%). The median wound length was 8 cm, and the median operation time was 230 min. Sublobar resection was performed in 26 patients (6%), and lobectomy or more was performed in 437 patients (94%). The patients’ postoperative complications were classified as Clavien–Dindo grade 0 in 340 patients(73.4%), grade I in 20 (4.3%), grade II in 45 (9.7%), grade IIIa in 53 (11.4%), and grade IIIb in 5 (1.2%). The median postCRP was 13.39 ng/ml. The perioperative factors of the VATS and thoracotomy groups are shown in Table 2. VATS was associated with a significantly shorter wound length (*p* <  0.01) and operation time (p <  0.01), and lower postCRP level (p <  0.01) in comparison to thoracotomy.

**Table 2:** The differences of perioperative factors by operative approach

Variables	VATS	Thoracotomy	*P* value
Wound length	7 (3–18)	20 (8–25)	< 0.01
Operation time	203 (67–674)	320 (154–672)	< 0.01
Postoperative CRP	12.2 (0.66–36.28)	20.58 (6.4–33.72)	< 0.01

*VATS* video-assisted thoracic surgery, *CRP* C-reactive protein

### Pathological factors

The pStage was IA in 225 patients (48.5%), IB in 101 (21.8%), IIA in 48 (10.4%), IIB in 43 (9.3%), and IIIA in 46 (10%). Adenocarcinoma was diagnosed in 350 patients (76%), squamous cell carcinoma was diagnosed in 91 patients (19.6%), and other types of lung cancer (adenosquamous cell carcinoma, pleomorphic carcinoma, large cell neuroendocrine carcinoma, and large cell carcinoma) were diagnosed in 22 patients (4.4%). The grade of differentiation was grade 1 (G1) in 173 patients (37.4%), grade 2 (G2) in 203 (43.8%), grade 3 (G3) in 75 (16.2%), and grade 4 (G4) in 12 82.6%). Ly was present in 192 patients (41.5%), and V was present in 225 patients (49%).

#### Correlation coefficients

The correlation coefficients are shown in Table 3. Positive correlations were observed between wound length and postCRP level (correlation coefficient; r = 0.39, *p* <  0.01), between wound length and operation time (r = 0.43, p <  0.01), and between operation time and postCRP level (r = 0.54, *p* <  0.03).

**Table 3:** Correlation coefficient

Variable	Correlation coefficient	*P* value
Wound length vs Postoperative CRP	0.399	< 0.01
Operation time vs Postoperative CRP	0.544	< 0.01
Operation time vs Wound length	0.432	< 0.01

*CRP* C-reactive protein

### Cutoff values calculated from ROC curves

The cutoff values of factors associated with recurrence were calculated by an ROC curve analysis. The following cutoff values were determined: operation time, 248 min; wound length, 10 cm; preCRP level, 0.14 ng/ml; and postCRP level, 14.49 ng/ml.

### Survival analyses

**Fig. 1:**
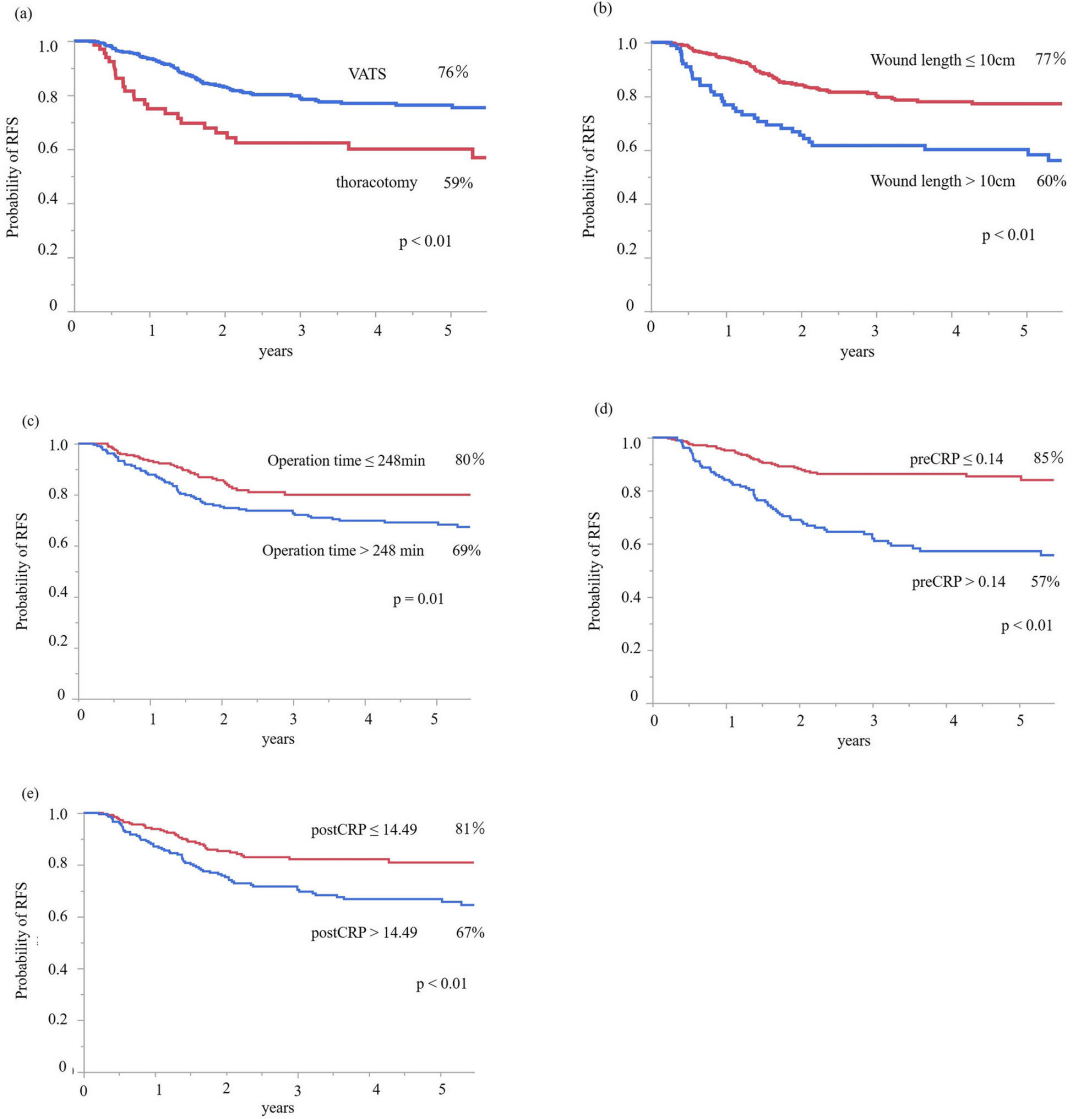
**a** Relapse-free survival rate in the VATS group was significantly longer than in the thoracotomy group. **b** Relapse-free survival in patients with a wound length of ≤10 cm was significantly longer than that in patients with a wound length of > 10 cm. **c** Relapse-free survival in patients with an operation time of ≤248 min was significantly longer than that in patients with an operation time of > 248 min. **d** Relapse-free survival in patients with a preCRP level of ≤0.14 was significantly longer than that in patients with a preCRP level of > 0.14. **e** Relapse-free survival in patients with a postCRP level of ≤14.49 was significantly longer than that in patients with a postCRP level of > 14.49

**Fig. 2:**
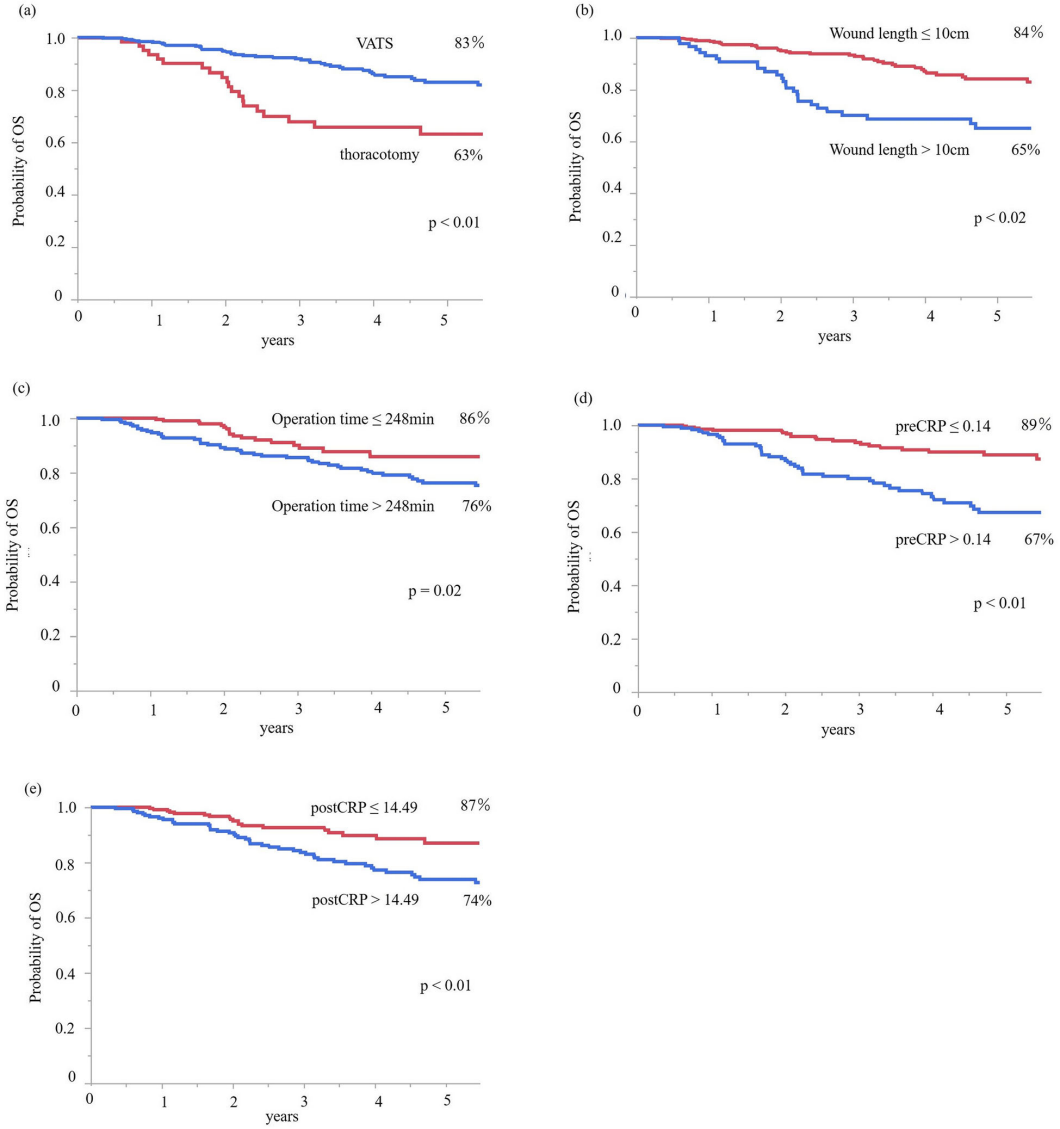
**a** Overall survival rate in the VATS group was significantly longer in comparison to the thoracotomy group. **b** Overall survival in the wound length ≤ 10 cm group was significantly longer than that in the wound length >  10 cm. **c** Overall survival in the operation time ≤ 248 min group was significantly longer than that in the operation time >  248 min group. **d** Overall survival in the preCRP ≤0.14 group was significantly longer than that in the preCRP > 0.14 group. **e** Overall survival in the postCRP ≤14.49 group was significantly longer than that in the postCRP > 14.49 group

RFS is shown in Fig. 1. There were significant prognostic differences according to the operative approach (*p* <  0.01), wound length (p <  0.01), operation time (*p* = 0.01), preCRP level (*p* <  0.01), and postCRP level (p <  0.01). OS is shown in Fig. 2. There were significant prognostic differences according to the operative approach (*p* <  0.01), wound length (*p* <  0.01), operation time (*p* = 0.02), preCRP level (*p* <  0.01), and postCRP level (*p* <  0.01).

### Univariate and multivariate analyses

The univariate and multivariate analyses of factors associated with RFS are summarized in Table 4. Sex (hazard ratio [HR], 1.92; *p* <  0.01), smoking history (HR, 2.10; *p* <  0.01), CEA (HR, 1.98; p <  0.01), operative approach (HR, 0.58; p <  0.01), wound length (HR, 2.07; p <  0.01), operation time (HR, 1.62; *p* = 0.01), operative procedure (HR, 1.99; *p* = 0.04), differentiation (HR, 2.09; *p* <  0.01), Ly (HR, 2.89; p <  0.01), V (HR, 3.26; p <  0.01), pStage (HR, 4.59; p <  0.01), preCRP level (HR, 2.80; p <  0.01), and postCRP level (HR, 1.89; *p* <  0.01) were identified as significant prognostic factors in the univariate analysis. CEA (HR, 1.58; *p* = 0.02), V (HR, 1.95; p = 0.01), pStage (HR, 2.57; *p* <  0.01), and preCRP level (HR, 1.91; p <  0.01) were identified as significant prognostic factors in the multivariate analysis.

**Table 4:** Cox proportional hazard analyses for factors affecting relapse free survival

		Univariate analysis		Multivariate analysis	
Variables		HR (95%CI)	*p*-value	HR (95%CI)	*p*-value
Gender	female	1			
	male	1.92 (1.27–2.99)	< 0.01		
Age	< 70y	1			
	≥ 70y	1.11 (0.75–1.63)	0.57		
Charlson comorbidity index	0–2	1			
	3–4	1.29 (0.41–7.85)	0.70		
Smoking status	never	1			
	Former / current	2.10 (1.38–3.28)	< 0.01		
CEA	≤ 5 ng/ml	1		1	
	> 5 ng/ml	1.98 (1.36–2.89)	< 0.01	1.58 (1.05–2.38)	0.02
Operative approach	Thoracotomy	1			
	VATS	0.57 (0.33–0.82)	< 0.01		
Wound length	≤ 10 cm	1			
	> 10 cm	2.07 (1.37–3.08)	< 0.01		
Operation time	≤ 248 min	1			
	> 248 min	1.62 (1.09–2.44)	< 0.01		
Operative procedure	Seg / Lob	1			
	Bilob / Pneumo	1.99 (1.01–3.55)	0.04		
pStage	I	1		1	
	II - IIIA	4.59 (3.13–6.78)	< 0.01	2.57 (1.60–4.18)	< 0.01
Histology	Ad	1			
	Non-Ad	1.32 (0.86–1.97)	0.19		
Differentiation	G1	1			
	G2 – G4	2.09 (1.38–3.24)	< 0.01		
Ly	absent	1			
	present	4.38 (2.25–9.01)	< 0.01		
V	absent	1		1	
	present	3.26 (2.16–5.05)	< 0.01	1.95 (1.15–3.38)	0.01
preCRP	≤ 0.14	1		1	
	> 0.14	2.80 (1.91–4.17)	< 0.01	1.91 (1.22–3.00)	< 0.01
postCRP	≤ 14.49	1			
	> 14.49	1.83 (1.24–2.72)	< 0.01		
Clavien-Dindo grade	0 - I	1			
	II - IIIb	1.36 (0.86–2.09)	0.17		

*CEA* carcinoembryonic antigen, *VATS* video-assisted thoracic surgery, *Seg* segmentectomy, *Lob* lobectomy, *Bilob* bilobectomy, *Pneumo* pneumonectomy, *pStage* pathological stage,

*Ly* lymphatic invasion, *V* vascular invasion, *preCRP* preoperative C-reactive protein, *postCRP* postoperative C-reactive protein

The results of the univariate and multivariate analyses of factors associated with OS are summarized in Table 5. Sex (HR, 3.32; *p* <  0.01), smoking history (HR, 4.12; p <  0.01), CEA (HR, 1.88; p <  0.01), operative approach (HR, 0.45; *p* <  0.01), wound length (HR, 2.41 *p* <  0.01), operation time (HR, 1.84 *p* = 0.02), operative procedure (HR, 2.98; p <  0.01), differentiation (HR, 1.92; p <  0.01), Ly (HR, 1.95 p <  0.01), V (HR, 3.36; p <  0.01), pStage (HR, 4.83; p <  0.01), preCRP level (HR, 2.85; *p* <  0.01), and postCRP level (HR, 2.20; *p* <  0.01) were identified as significant prognostic factors in the univariate analysis. Smoking history (HR, 2.36; *p* = 0.03), V (HR, 2.80; *p* <  0.01), and pStage (HR, 3.26; *p* <  0.01) were identified as significant prognostic factors in the multivariate analysis.

**Table 5:** Cox proportional hazard analyses for factors affecting overall survival

		Univariate analysis		Multivariate analysis	
Variables		HR (95%CI)	*p*-value	HR (95%CI)	*p*-value
Gender	female	1			
	male	3.32 (1.89–6.31)	< 0.01		
Age	< 70y	1			
	≥ 70y	1.08 (0.67–1.70)	0.74		
Charlson comorbidity index	0–2	1			
	3–4	1.37 (0.22–4.38)	0.67		
Smoking status	never	1		1	
	Former /current	4.12 (2.31–8.03)	< 0.01	2.36 (1.07–5.56)	0.03
CEA	≤ 5 ng/ml	1			
	> 5 ng/ml	1.88 (1.20–2.95)	< 0.01		
Operative approach	Thoracotomy	1			
	VATS	0.45 (0.28–0.75)	< 0.01		
Wound length	≤ 10 cm	1			
	> 10 cm	2.41 (1.51–3.79)	< 0.01		
Operation time	≤ 248 min	1			
	> 248 min	1.84 (1.09–3.23)	0.02		
Operative procedure	Seg / Lob	1			
	Bilob / Pneumo	2.98 (1.48–5.41)	< 0.01		
pStage	I	1		1	
	II - IIIA	4.83 (3.05–7.82)	< 0.01	3.26 (1.77–6.08)	< 0.01
Histology	Ad	1			
	Non-Ad	1.60 (0.98–2.55)	0.05		
Differentiation	G1	1			
	G2 – G4	1.92 (1.19–3.17)	< 0.01		
Ly	absent	1			
	present	1.95 (1.24–3.11)	< 0.01		
V	absent	1		1	
	present	3.36 (2.06–5.69)	< 0.01	2.80 (1.49–5.38)	< 0.01
preCRP	≤ 0.14	1			
	> 0.14	2.85 (1.80–4.61)	< 0.01		
postCRP	≤ 14.49	1			
	> 14.49	2.20 (1.35–3.69)	< 0.01		
Clavien-Dindo grade	0 - I	1			
	II - IIIb	1.45 (0.83–2.43)	0.17		

*CEA* carcinoembryonic antigen, *VATS* video-assisted thoracic surgery, *Seg* segmentectomy, *Lob* lobectomy, *Bilob* bilobectomy, *Pneumo* pneumonectomy, *pStage* pathological stage,

*Ly* lymphatic invasion, *V* vascular invasion, *preCRP* preoperative C-reactive protein, *postCRP* postoperative C-reactive protein

### Sub-analyses

The univariate and multivariate analyses of factors associated with RFS for pathological stage I are summarized in Table 6 (Supplemental 1). Sex (HR, 2.49; *p* <  0.01), smoking history (HR, 3.19; p <  0.01), CEA (HR, 2.08; *p* = 0.01), histology (HR, 2.01; *p* = 0.03), differentiation (HR, 4.16; *p* <  0.01), Ly (HR, 2.13 p = 0.01), V (HR, 2.99; *p* <  0.01), and preCRP level (HR, 3.17; *p* <  0.01) were identified as significant prognostic factors in the univariate analysis. Differentiation (HR, 2.33; p = 0.03) and preCRP (HR, 3.08; *p* <  0.01) were identified as significant prognostic factors in the multivariate analysis.

The univariate and multivariate analyses of factors associated with OS for pathological stage I are summarized in Table 7 (Supplemental 2). Sex (HR, 6.53; *p* <  0.01), smoking history (HR, 11.92; p <  0.01), histology (HR, 2.67; *p* = 0.01), differentiation (HR, 3.66; p <  0.01), V (HR, 3.97; p <  0.01), and preCRP level (HR, 3.07; p <  0.01) were identified as significant prognostic factors in the univariate analysis. Smoking status (HR, 9.05; p <  0.01), V (HR, 3.20; p = 0.01) and preCRP (HR, 2.21; *p* = 0.04) were identified as significant prognostic factors in the multivariate analysis.

## Discussion

We showed that VATS was associated with a significantly shorter wound length and operation time, and lower postCRP level in comparison to thoracotomy in patients who underwent surgery for NSCLC. VATS has been reported to be associated with less pain, a shorter hospital stay and a reduced cytokine response in comparison to thoracotomy [4–10]. CRP and interleukin (IL)-6 have often been analyzed as parameters of operative invasiveness, and in patients who undergo VATS, these values are significantly lower in comparison to those who undergo thoracotomy [8–10]. In the present study, the operation time and wound length were positively correlated with the postCRP level; thus, it was suggested that the operation time and wound length are associated with operative invasiveness and that VATS is less invasive approach.

The relationship between operative invasiveness, including the postoperative inflammatory response, and the prognosis of NSCLC patients who undergo surgery has not been clear. VATS has been reported to result in reduced postoperative CRP and IL-6 levels in comparison to thoracotomy [8–10, 13–16]. IL-6 has been reported to promote cell proliferation [17–20], affect insulin growth factor (IGF), and result in insulin growth factor binding protein (IGFBP) 3 inhibition, thereby contributing to an environment favoring tumor proliferation in patients with NSCLC [21, 22]. Although some reports have demonstrated a prognostic advantage of VATS in comparison to thoracotomy, it was considered that the prognostic advantage was influenced by a patient bias [23, 24]. The present study also demonstrated that the DFS and OS of the VATS group were better than those in the thoracotomy group; however, a multivariate analysis of DFS and OS confirmed the non-superiority of VATS. Because other reports demonstrated that long-term survival after VATS is not inferior in comparison to thoracotomy, it might be revealed that a less invasive operative approach itself is not a prognostic factor [24–27].

The present study identified the preCRP level as a significant prognostic factor for recurrence in patients who received surgery. Previous studies reported that the CRP level is an important prognostic factor in NSCLC patients [28–30]. Several mechanisms for the elevation of the CRP level in cancer patients have been proposed. First, tumor growth might cause tissue inflammation and CRP elevation [31, 32]. Second, CRP might be an indicator of the immune response to tumor antigens [33–35]. Third, there is evidence that cancer cells increase the production of inflammatory proteins such as CRP. Based on these mechanisms, patients with high preCRP levels might already have a cytological tumor spread that cannot be detected by either imaging studies or pathologic examinations. Furthermore, circulating cancer cells might be responsible for early recurrence in patients with high preCRP levels. A previous study demonstrated that CRP is positively correlated with the pathological tumor size and pathological stage in NSCLC patients [28]; thus, the preCRP level might be a prognostic indicator for NSCLC patients who have undergone surgery.

The present study was associated with several limitations. First, the study was retrospective in nature, and potentially involved unobserved cofounding and selection biases. Second, the present study was performed at a single institution, and the study population was relatively small.

## Conclusions

Our findings suggested that operation time and wound length reflect operative invasiveness and that VATS is a less invasive approach for NSCLC patients who have undergone surgery. Although VATS itself might be not a prognostic factor, the preCRP level might be a prognostic factor in NSCLC patients who have undergone surgery.

## Supplementary information

**Additional file 1.**

**Additional file 2.**

## Data Availability

The data sets supporting the conclusions of the present study are included in this published article.

## Abbreviations

NSCLC: Non-small cell lung cancer
postCRP: postoperative C-reactive protein
VATS: Video-assisted thoracic surgery
CC: Correlation coefficient
HR: Hazard ratio
pStage: pathological stage
preCRP: preoperative CRP level
APPs: Acute-phase proteins
CEA: Carcinoembryonic antigen
C-VATS: Complete VATS
H-VATS: Hybrid VATS
Ly: Lymphatic invasion
V: Vascular invasion
RFS: Relapse-free survival
OS: Overall survival
IL: Interleukin
IGF: Insulin growth factor
IGFBP: Insulin growth factor binding protein
