# Supplementary material for: Operative invasiveness does not affect the prognosis of patients with non-small cell lung cancer
Source: BMC Pulm Med. 2020 Oct 15;20:265. doi: 10.1186/s12890-020-01264-x (PMC7558745; doi:10.1186/s12890-020-01264-x)
Supplement: Supplementary file 1 — Additional file 1. [file 12890_2020_1264_MOESM1_ESM.docx]

Table 6. Cox proportional hazard analyses for factors affecting relapse free survival in pathological stage I

|  | Univariate analysis | | |  | Multivariate analysis | |
| --- | --- | --- | --- | --- | --- | --- |
| Variables |  | HR (95%CI) | p-value |  | HR (95%CI) | p-value |
| Gender | female | 1 |  |  |  |  |
|  | male | 2.49 (1.30 – 5.17) | < 0.01 |  |  |  |
| Age | < 70y | 1 |  |  |  |  |
|  | ≥ 70y | 1.56 (0.86 – 2.84) | 0.13 |  |  |  |
| Charlson comorbidity index | 0 - 2 | 1 |  |  |  |  |
|  | 3 - 4 | 0.98 (0.05 – 4.56) | 0.98 |  |  |  |
| Smoking status | never | 1 |  |  |  |  |
|  | Former / current | 3.19 (1.63 - 6.82) | < 0.01 |  |  |  |
| CEA | ≤ 5 ng/ml | 1 |  |  |  |  |
|  | > 5 ng/ml | 2.08 (1.12 – 3.77) | 0.01 |  |  |  |
| Operative approach | Thoracotomy | 1 |  |  |  |  |
|  | VATS | 0.79 (0.33 - 2.33) | 0.64 |  |  |  |
| Wound length | ≤ 10 cm | 1 |  |  |  |  |
|  | > 10 cm | 1.34 (0.57 - 2.77) | 0.46 |  |  |  |
| Operation time | ≤ 248 min | 1 |  |  |  |  |
|  | > 248 min | 1.26 (0.66 - 2.42) | 0.47 |  |  |  |
| Operative procedure | Seg / Lob | 1 |  |  |  |  |
|  | Bilob / Pneumo | 1.39 (0.72 - 3.55) | 0.33 |  |  |  |
| Histology | Ad | 1 |  |  |  |  |
|  | Non-Ad | 2.01 (1.05 - 3.70) | 0.03 |  |  |  |
| Differentiation | G1 | 1 |  |  | 1 |  |
|  | G2 – G4 | 4.16 (2.11 – 8.96) | < 0.01 |  | 2.33 (1.05-5.58) | 0.03 |
| Ly | absent | 1 |  |  |  |  |
|  | present | 2.13 (1.16 – 3.87) | 0.01 |  |  |  |
| V | absent | 1 |  |  |  |  |
|  | present | 2.99 (1.64 - 5.61) | < 0.01 |  |  |  |
| preCRP | ≤ 0.14 | 1 |  |  | 1 |  |
|  | > 0.14 | 3.17 (1.74 - 5.88) | < 0.01 |  | 3.08 (1.63 – 5.95) | < 0.01 |
| postCRP | ≤ 14.49 | 1 |  |  |  |  |
|  | > 14.49 | 1.70 (0.93 – 3.19) | 0.08 |  |  |  |
| Clavien-Dindo grade | 0 - I | 1 |  |  |  |  |
|  | II - IIIb | 1.17 (0.52 - 2.35) | 0.67 |  |  |  |

CEA: carcinoembryonic antigen, VATS; video-assisted thoracic surgery, Seg; segmentectomy, Lob; lobectomy, Bilob; bilobectomy, Pneumo; pneumonectomy, Ly; lymphatic invasion,

V; vascular invasion, preCRP; preoperative C-reactive protein, postCRP; postoperative C-reactive protein.
