# Supplementary material for: Operative invasiveness does not affect the prognosis of patients with non-small cell lung cancer
Source: BMC Pulm Med. 2020 Oct 15;20:265. doi: 10.1186/s12890-020-01264-x (PMC7558745; doi:10.1186/s12890-020-01264-x)
Supplement: Supplementary file 2 — Additional file 2. [file 12890_2020_1264_MOESM2_ESM.docx]

Table 7. Cox proportional hazard analyses for factors affecting overall survival in pathological stage I

|  | Univariate analysis | | |  | Multivariate analysis | |
| --- | --- | --- | --- | --- | --- | --- |
| Variables |  | HR (95%CI) | p-value |  | HR (95%CI) | p-value |
| Gender | female | 1 |  |  |  |  |
|  | male | 6.53 (2.28 - 27.48) | < 0.01 |  |  |  |
| Age | < 70y | 1 |  |  |  |  |
|  | ≥ 70y | 1.61 (0.75 – 3.48) | 0.21 |  |  |  |
| Charlson comorbidity index | 0 - 2 | 1 |  |  |  |  |
|  | 3 - 4 | 1.86 (0.10 - 8.93) | 0.57 |  |  |  |
| Smoking status | never | 1 |  |  | 1 |  |
|  | Former /current | 11.92 (3.55 - 74.13) | < 0.01 |  | 9.05 (1.70 - 72.95) | < 0.01 |
| CEA | ≤ 5 ng/ml | 1 |  |  |  |  |
|  | > 5 ng/ml | 1.58 (0.69 – 3.41) | 0.25 |  |  |  |
| Operative approach | Thoracotomy | 1 |  |  |  |  |
|  | VATS | 0.65 (0.24 - 2.27) | 0.46 |  |  |  |
| Wound length | ≤ 10 cm | 1 |  |  |  |  |
|  | > 10 cm | 1.20 (0.39 - 2.96) | 0.71 |  |  |  |
| Operation time | ≤ 248 min | 1 |  |  |  |  |
|  | > 248 min | 1.41 (0.60 - 3.56) | 0.42 |  |  |  |
| Operative procedure | Seg / Lob | 1 |  |  |  |  |
|  | Bilob / Pneumo | 1.57 (0.77 - 4.23) | 0.51 |  |  |  |
| Histology | Ad | 1 |  |  |  |  |
|  | Non-Ad | 2.67 (1.20 - 5.71) | 0.01 |  |  |  |
| Differentiation | G1 | 1 |  |  |  |  |
|  | G2 – G4 | 3.66 (1.61 - 9.38) | < 0.01 |  |  |  |
| Ly | absent | 1 |  |  |  |  |
|  | present | 1.40 (0.61 - 3.03) | 0.40 |  |  |  |
| V | absent | 1 |  |  | 1 |  |
|  | present | 3.97 (1.82 - 9.30) | < 0.01 |  | 3.20 (1.25 - 8.79) | 0.01 |
| preCRP | ≤ 0.14 | 1 |  |  | 1 |  |
|  | > 0.14 | 3.07 (1.43 - 6.81) | < 0.01 |  | 2.21 (1.00-5.06) | 0.04 |
| postCRP | ≤ 14.49 | 1 |  |  |  |  |
|  | > 14.49 | 2.00 (0.91 – 4.72) | 0.08 |  |  |  |
| Clavien-Dindo grade | 0 - I | 1 |  |  |  |  |
|  | II - IIIb | 1.74 (0.67 - 3.98) | 0.23 |  |  |  |

CEA: carcinoembryonic antigen, VATS; video-assisted thoracic surgery, Seg; segmentectomy, Lob; lobectomy, Bilob; bilobectomy, Pneumo; pneumonectomy, Ly; lymphatic invasion,

V; vascular invasion, preCRP; preoperative C-reactive protein, postCRP; postoperative C-reactive protein.
